# Circadian Cycles of Gene Expression in the Coral, *Acropora millepora*

**DOI:** 10.1371/journal.pone.0025072

**Published:** 2011-09-19

**Authors:** Aisling K. Brady, Kevin A. Snyder, Peter D. Vize

**Affiliations:** Department of Biological Sciences, University of Calgary, Calgary, Alberta, Canada; Vanderbilt University, United States of America

## Abstract

**Background:**

Circadian rhythms regulate many physiological, behavioral and reproductive processes. These rhythms are often controlled by light, and daily cycles of solar illumination entrain many clock regulated processes. In scleractinian corals a number of different processes and behaviors are associated with specific periods of solar illumination or non-illumination—for example, skeletal deposition, feeding and both brooding and broadcast spawning.

**Methodology/Principal Findings:**

We have undertaken an analysis of diurnal expression of the whole transcriptome and more focused studies on a number of candidate circadian genes in the coral *Acropora millepora* using deep RNA sequencing and quantitative PCR. Many examples of diurnal cycles of RNA abundance were identified, some of which are light responsive and damped quickly under constant darkness, for example, *cryptochrome 1* and *timeless*, but others that continue to cycle in a robust manner when kept in constant darkness, for example, *clock*, *cryptochrome 2*, *cycle* and *eyes absent*, indicating that their transcription is regulated by an endogenous clock entrained to the light-dark cycle. Many other biological processes that varied between day and night were also identified by a clustering analysis of gene ontology annotations.

**Conclusions/Significance:**

Corals exhibit diurnal patterns of gene expression that may participate in the regulation of circadian biological processes. Rhythmic cycles of gene expression occur under constant darkness in both populations of coral larvae that lack zooxanthellae and in individual adult tissue containing zooxanthellae, indicating that transcription is under the control of a biological clock. In addition to genes potentially involved in regulating circadian processes, many other pathways were found to display diel cycles of transcription.

## Introduction

Biological clocks regulate many diverse aspects of animal behavior and physiology. Light plays a major role in entraining most biological clocks operating on a daily cycle, but other factors, such as nutrient intake, can also drive clocks (e.g. [1], [2]). Like other animals, corals exhibit major transitions between daytime and nighttime. For example, in daylight most scleractinian corals retract their tentacles and rely on photosynthesis within endosymbiotic zooxanthallae to produce energy. At night, when no photosynthesis occurs, corals extend tentacles and actively feed on drifting prey [3]. Like many daily cycles, the extension and retraction of tentacles is an entrained biological process that continues in a rhythmic manner in corals that are kept in constant darkness [4].

Other processes in corals may also be under the control of biological clocks, for example the carefully controlled timing of gamete/planula release during sexual reproduction (e.g. [5]–[7]) and various metabolic processes [8]. In an effort to shed light on circadian processes in corals, we and others have previously searched in the coral transcriptome for potential orthologs of genes involved in regulating circadian processes in other animals [9]–[11]. Candidate orthologs were identified for many such genes, including *bmal/cycle*, *clock*, *cryptochromes 1* and *2*, *nr1d1*, *period 1* and *2*, *timeless* and many others, some of which have also been described in the sea anemone *Nematostella vectensis* [10], [11]. Photoreceptors that may synchronize light mediated responses have also been described in corals [10], [12].

There is an important difference between daily cycles that respond directly to light, for example photoreception, and process that are entrained by light but continue to cycle in a circadian manner when kept in constant darkness. The former type of process responds directly to light, while the latter are under the control of an entrained clock [13]. Direct responses are sometimes said to be controlled by an hour-glass mechanism while entrained processes are described as being controlled by a biological clock or a circadian mechanism. It is the entrained processes that play central roles in regulating circadian cycles [14].

In this study we explore cyclic patterns of transcription in the coral, *Acropora millepora* and whether such patterns are direct responses to light or are controlled by a biological clock. These analyses are preformed in both populations of azooxanthellar 7 day old larvae by deep sequencing and by quantitative PCR (QPCR), and in individual zooxanthellar adult tissue by QPCR. Bioinformatic analysis of *A. millepora* transcriptome sequencing shows that most, but not all, of the candidate circadian orthologs display strong diurnal regulation, with up to 200 fold higher levels of expression between different light conditions. Thousands of other genes also display differences in transcription in response to light. More detailed analysis of transcription profiles over a 24 hour period demonstrated that many patterns are, as predicted, rhythmic, and continue to cycle for at least 24 hours when corals are kept in total darkness.

## Methods

### Sample Collection

In November 2008, adult colonies of *A. millepora* were collected from Cattle Bay at Orpheus Island (18°35′53.4″S 146°29′28.8″E) and Pelorus Island (18°33′40.6″S 146°30′03.03″E), Great Barrier Reef, Australia. Individual colonies were placed in separate bins back at the Orpheus Island Research Station, and colonies spawned at similar times as their species cohort on the reef. Gamete bundles from more than 10 adult colonies of *A. millepora* were mixed and kept undisturbed for approximately 1–2 hours to allow cross-fertilization. Newly fertilized larvae were washed and transferred to two fiberglass 500 L larval culture tanks continuously receiving 28°C 1.0 µm filtered seawater. Each tank was exposed to 12 hours light and 12 hours dark (12∶12 LD) for six days. Light intensity was measured to be 150 lux under 40 watt white fluorescent lights (40 Watt Sun-Glo fluorescent bulbs, Hagen). On the seventh day, one tank continued the 12∶12 LD treatment, while the other was completely darkened for a 24-hour continuous dark treatment (12∶12 DD). Larvae were collected every four hours, beginning two hours after initial lights on (and subjective lights on for DD tank) for a 24-hour period. The larvae were sieved using a fine nitex-mesh filter and preserved using a commercial RNA storage product from Ambion (cat. no. AM7024). 200 to 500 larvae were collected per sample. Dark samples were collected using a minimal exposure to red light from a red LED head lamp (Energizer HDL33AODE) and transferred immediately into RNA storage solution and frozen. This procedure was repeated in November, 2009, with a second batch of similarly produced larvae.

Adult colonies collected from Cattle Bay, Orpheus Island in November 2009, were also exposed to a 13∶11 hour LD (light∶dark) treatment for 35 days in an experimental setting. Light intensity was much higher at ∼21,000 lux using full spectrum white lamps (Sylvania Coral-Arc lamps HS1-TD-150 Watt, 20,000K). Two individuals, 20 days apart, were sampled every four hours to determine 24 hour changes in gene expression. One two to three cm branch per sample was broken from the colony, immediately immersed in 1 mL of trizol reagent (Invitrogen), and ground into a slurry with a mortar and pestle. Samples were then stored at −80°C. All samples were transported to the laboratory on dry ice, under GBRMPA Collection Permit Number G09/31214.1 and CITES Export Number 2009-AU-563189.

### RNA Extraction

Total RNA was extracted following the trizol protocol provided by Invitrogen, after both adult and larval tissues were homogenized using disposable pestles. Samples were further purified by a DNase1 digestion, a phenol-chloroform extraction, potassium-acetate and ethanol precipitation, and two washes in 75% ethanol. RNA pellets were redissolved in RNase-free water. RNA quantity was determined with a NanoDrop ND-1000 spectrophotomoter.

### Deep Sequencing and Sequence Analysis

Two larval samples exposed to the 12∶12 LD experimental treatment in 2008 were selected for next generation whole transcriptome sequencing. These samples were 12 hours apart in sampling time, with the day sample collected 10 hours after initial lights on and the night sample at 10 hours after lights off (22 hours after initial lights on). The two samples were sent to the BC Cancer Agency Genome Sciences Centre, Vancouver, Canada, for cDNA library development and Illumina Genome Analyzer Solexa whole transcriptome sequencing. The output from this data included 10 million reads for 5′ and 3′ ends, per sample. Public access to this data set is available at ftp://ftp.xenbase.org/pub/Coral/.

Sequences from both sample outputs were compared against the annotated *A. millepora* 454 and Expressed Sequence Tag (EST) transcriptome gene set generated by Meyer et al. [15], via BLAST. The BLAST analysis was optimized for highly similar sequences (MegaBLAST, [16]), with a minimum e-value cut-off set to 1e-10. The e-value is a score that indicates how similar two sequences are to one another, with lower values indicating higher similarity. The BLAST analysis was analyzed within custom perl-scripts that counted the number of sequences matching the transcriptome gene set for each sequence in either day and night samples. The perl-scripts are available upon request.

The sequencing output was further analyzed for similar biological functions, based on Gene Ontology (GO) terms, through the Gene Set Enrichment Analysis (GSEA) software, Version 2.06, supplied by the Broad Institute [17], [18]. Three files were created: i) Gene Cluster Text file, listing coral contig identification numbers and transcript counts for day and night (sum of 5′ and 3′ sequence reads for each experimental condition); ii) Gene Matrix Transposed file, listing each *Nematostella vectensis* GO term and all coral contigs identified by BLAST; and iii) Categorical Class File, defining the class or template labels associated with each sample in the expression data. The number of permutations was set to 100, the maximum number of coral contigs mapped to a GO term was set to 2,000 while the minimum was set to 1. The Enrichment Score, which reflects the degree to which a gene set is over-represented at the top or bottom of a ranked list of genes, had the P-value set to 1.

### Reverse Transcription and QPCR

50 µL of cDNA was synthesized from 500 ng of RNA by reverse transcription. 10 U µL^−1^ AMV Reverse Transcriptase was used according to instructions by the supplier (NEB), with an oligo (dT) primer_12–18_ (Invitrogen).

QPCR was used to identify relative changes in gene expression over the complete 24 hour sampling period, as well as identify whether candidate circadian genes were under the control of an entrained biological clock or if they were regulated directly by light. Candidate genes were selected based on results from Vize [10], and primers were developed with Primer3 Software [19]. Primer specifications were set to generate 100–150 bp amplicons, have an optimal size of 20 bases, a range of melting temperature from 55–61°C, and a primer GC content ranging from 40–55% (see Table S1 for primer sequences). One µL of cDNA was used in triplicate 20 µL qPCR reactions, with 1 µM primers (1.5 µL forward, 1.5 µL reverse), 6 µL nuclease-free water, and 10 µL SYBR green with fluorescein mix (Quantace) for 40 cycles on a BioRad iCycler iQ Real Time PCR System. Cycle threshold values for each time point, in triplicate, were compared to the internal reference genes RNA polymerase II (RPII) and adenosyl-homocysteinase (AdoH), according the 2^−ΔΔC^_T_ method [20], with 10 hours after lights on (or subjective lights on) acting as the time point for comparison. Amplification efficiencies were conducted using the methods described in Livak and Schmittgen [20]. A two-fold serial dilution series was used, starting with 2000 ng RNA equivalent cDNA from a separate *A. millepora* laval sample, and ending with 62.5 ng RNA equivalent cDNA. A plot of log cDNA dilution versus ΔC_T_ (C_T,gene_ - C_T,RefGene_) was made to ensure the absolute value of the slope was ≤0.1. The results using AdoH as the internal reference gene are not presented, as they did not differ significantly from RPII.

### Graphing and Statistical Analyses

QPCR data was analyzed through both graphical interpretation and statistical analysis. Graphs were created using GraphPad Prism Software, Version 5.0. Mean (± SEM) of the triplicate fold change in mRNA expression levels were graphed using the XY scatterplot function. Statistical analyses were performed using JMP Statistical Software Package, Version 8.0 Data were checked for normal distributions using the Shapiro-Wilk W Goodness of Fit test. Data with p-values≥0.02 were classified as normally-distributed. Box-Cox transformations were performed to make data normal where necessary, and analyses were conducted on transformed data (best transformations were used). Data was also confirmed to have equal variances, using the Levene test. Standard least squares multiple regressions were used to identify significant interactions between light treatment (LD vs. DD) and time. One-way ANOVAs were used to determine differences between light treatments, and was followed with Bonferroni Post-Tests for differences at each time point. Presented analyses are for the 2009 data, with similar statistical results confirmed in replicate samples.

## Results

RNA was isolated from *Acropora millepora* larvae at 4 hour intervals over a 24 hour period, beginning at 7 days of culture post-fertilization. At this stage of development larvae are beginning to transition from the free swimming elongated planulae form to a pelagic pre-metamorphic morphology, with the beginning of tentacle buds just beginning to appear. Larvae were collected from two different mass culture tanks, one kept on a continuous 12∶12 light∶dark cycle for all 7 days (LD sample) and the a second tank that was kept on a 12∶12 LD cycle for 6 days, then kept in constant darkness for the 24 hour sampling period (DD sample).

**Figure 1:**
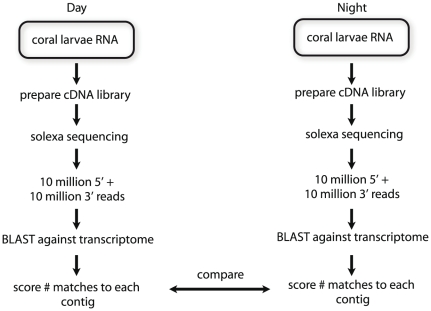
Solexa sequencing data processing pipeline. Detailed steps involved in Solexa deep sequencing of coral larvae samples for both day and night (12 hour difference). Preparation of cDNA library, sequencing and generation of output were performed by the BC Genome Sciences Centre, while all other components were performed by authors.

To determine which *Acropora* genes display diurnal patterns of transcription, we preformed Solexa sequencing of LD samples collected in the late day or late night. A total of 20 million reads was generated from each RNA sample and processed via the pipeline illustrated in Figure 1. The target transcriptome contained 40,000 contigs, representing approximately 3× coverage of the transcriptome, assembled from *Acropora millepora* EST and 454 reads by Meyer et al. [15]. Each sequence read was analyzed by BLAST [16] for matches to the target transcriptome with a cutoff of 1e−10. A custom perl script was then used to score the number of matches to each contig. This script is available on request.

Over 11,800 contigs had read numbers that differed by less than 20% between night and day samples. These serve as 11,800 controls for contigs that had different scores between the two samples. A 1.5 fold cut off difference is often used in genomics approaches, such as microarrays, as a threshold indicating functionally relevant differences in gene expression levels (e.g. [21]). Table 1 presents the number of BLAST matches to candidate circadian genes selected because of their involvement in regulating circadian cycles in other organisms. All of the candidate genes with one exception, *slmb* (*supernumerary limbs*), had greater than 1.5 fold differences in abundance between the day and the night samples. Two of these genes, *cryptochrome* 1 and 2 have been previously reported to display diurnal patterns of transcription in corals, with expression being higher in the light phase than in the dark [7], [8], [9]. Similar results were found in our sequencing data, with *cry1* having a 16.2 LD ratio and *cry2* having a 5.96 LD ratio. Two additional genes had stronger expression in the light sample, *clock* and *per1*, while two had stronger expression in the dark; *timeless* (LD ratio 0.39) and *vrille* (LD ratio 0.005). The *vrille* gene, a leucine-zipper class transcription factor essential for circadian rhythms in *Drosophila* [22], displayed an extraordinary response to the light cycle, with only 12 reads in the day sample but 2,499 reads in the night sample, a 200-fold difference (Table 1). Two additional genes that have recently been shown to participate in the seasonal responses to changes in day length are *eyes absent* (*eya*) and the *six homeobox* (*six*) [23], [24]. Both of these genes are transcription factors, and both display diurnal differences in expression in the sequence data, with *eya* having a LD ratio of 0.17 and *six* an LD of 0.59. *Slmb* (*supernumerary limbs*), had a 0.98 LD ratio with 39 and 40 matches respectively within light and dark samples, indicating that it does not respond to light. *Slmb* encodes an F-box protein that is necessary for correct phosphorylation, but not transcriptional cycling, of *period* and *timeless* genes in *Drosophila* [25]. Although slmb protein levels do cycle modestly in *Drosophila* under LD conditions [25], transcriptional oscillations have not been previously reported for this gene.

**Table 1:** Analysis of diurnal transcription using Solexa sequencing.

Gene	Day Reads	Night Reads	LD Ratio	NCBI
RNA polymerase II	745	750	0.99	EZ031385
adenosyl homocysteinase	3947	3920	1.01	EZ019649
cry1	5724	353	16.2	EZ010289
cry2	1843	309	5.96	EF202590
clock	681	216	3.15	EZ010226
vrille	12	2499	0.005	EZ035738
eya	4	23	0.17	EZ014333
timeless	27	69	0.39	EZ013923
six	345	589	0.57	EZ036950
cycle	6	10	0.6	EZ013275
slmb	39	40	0.98	EZ011919

The sequencing data was also searched for two additional types of information; genes that showed no diurnal differences for use as controls in later experiments, and clustering to search for groups of genes participating in coordinated pathways that are co-regulated. Two common control genes in gene expression studies are the translation factor *ef1a* and various actin isoforms. *Ef1a* is a very abundant mRNA (33,365 matches during the day and 30,896 during the night) and therefore not an ideal control. Cytoplasmic actin varies in abundance between day (27 matches) and night (66 matches) samples with a LD ratio of 0.41, and though commonly used as a control, may not be a good choice. Various other common control genes were therefore evaluated, and two, *RNA polymerase II* and *adenosyl homocysteinase*, were found to have very similar day∶night sequence read numbers, 0.99 and 1.01 respectively (Table 1). As the abundance of *RNA polymerase II* transcripts was similar to many of the circadian candidate genes this gene was used as the primary control, but all data were also secondarily confirmed using *adenosyl homocysteinase*.

### Pathway analysis

GO terms were assigned to *Acropora* contigs by identifying the closest blast hit to the machine annotated *Nematostella* genome. The number of sequence hits against each GO term in day and night samples was then analyzed using the GSEA software package (see Methods). This system identified 26 gene sets that were expressed at different levels in the two samples. Processes that differed between day and night samples included mitosis, mitochondrial energy production, cellular energy production and light associated processes such as retinal metabolic processes and *rhodopsin* gene expression. Selected examples of differently active processes in daytime samples are illustrated by Table 2, and the full analysis in supplemental data (Table S2).

**Table 2:** GSEA pathway analysis.

Gene set	Enrichment Score	P Value	Core Genes
DNA photolyase	1.58	0	4
mitochondrial respiratory chain	1.47	0	7
mitotic cell cycle checkpoint	1.42	0	1
histone acetyltransferase complex	1.42	0	1
neuropeptide signaling	1.39	0	16
generation of precursor metabolites and energy	1.34	0.08	4
regulation of mitosis	1.33	0.09	1
retinal metabolic processes	1.33	0.05	1
regulation of rhodopsin gene expression	1.32	0.1	1

### Quantitative expression analysis of candidate circadian genes in larva

**Figure 2:**
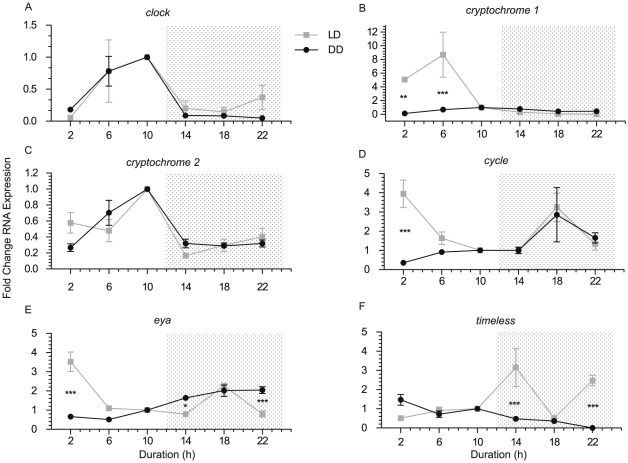
QPCR analysis of candidate circadian gene expression in larvae. Larvae were exposed to either a 12∶12 LD treatment or 12∶12 DD treatment for 24 hours, and were analyzed with QPCR for rhythmicity in candidate circadian genes (a. *clock*, b. *cryptochrome 1*, c. *cryptochrome 2*, d. *cycle*, e. *eyes absent*, f. *timeless*). Relative fold changes (mean ± SEM of triplicate QPCR reactions) in RNA expression levels are presented based on QPCR analysis of the 2009 larval sample. Shaded areas represent periods of darkness, with the exception of DD samples, which were darkened for 24 hours. Bonferroni post-tests were performed to confirm statistical significant differences at each time point. * represents P<0.05, ** represents P<0.01, and *** represents P<0.001.

In order to determine whether differences in levels of expression between samples collected from light or dark were under the control of an endogenous clock, QPCR was performed on RNA from two different sample sets, one collected following the 2008 spawn and used in deep sequencing (Table 1) and a sample collected after the 2009 spawning event (see Methods). Coral larvae were kept in duplicate tanks under a 12∶12 light∶dark (LD) regimen for 6 days followed by a 24 hour sampling period during which one tank continued to receive the 12∶12 LD regimen while the other was kept in total darkness. The time at which lights were activated in the morning is denoted as zero hours and lights were turned off at 12 hours (Figure 2). Periods of darkness are denoted in Figure 2 with a grey background.

#### Clock

This gene (NCBI EZ010226) displayed a diurnal transcription pattern. Deep sequencing found stronger *clock* expression in light (LD ratio 3.15) as did QPCR analysis of the same samples plus a second independent larval sample (Figure 2a). *Clock* transcription is strongly rhythmic in larvae and shows a similar pattern of expression under constant darkness as it does under a 12∶12 LD treatment. The LD and DD expression patterns were the same, indicating that expression is under the control of an endogenous biological clock.

#### Cryptochrome 1

*Cry1* (NCBI EZ010289) [9], annotated in the sea anemone *Nematostella* as *cry1a* [11], shows light responsive transcription (Figure 2b) but no clock driven expression, as has also previously been reported by Levy et al. [9] and Reitzel et al. [11]. The LD ratio via deep sequencing is very high, at 16.2, with highest expression in the day time. In all three larval samples examined expression is strongest in the middle of the light period, approximately 6 hours after lights on. The interaction of light treatment and time (i.e. how the light treatment varies across each time point) is significant(P<0.0001). Under constant darkness *cry1* expression remains low indicating that transcription is not under the control of an endogenous clock.

#### Cryptochrome 2

*Cry2* (NCBI EF202590, [7], [9], [10]), annotated as *Cry1b* in *Nematostella* [11], displayed strong expression in light (deep sequencing LD ratio of 5.96), and robust rhythmic cycling under constant darkness (Figure 2c). Expression peaked late in the day, consistent with the deep sequencing data. There was no difference between LD and DD samples, as expected for a gene whose transcription is regulated by an entrained biological clock.

#### Cycle/bmal

The *cycle* gene (NCBI EZ013275) was found to be expressed in a cyclic manner and displayed rhythmic transcriptional oscillations under constant darkness. Analysis of larval samples by QPCR and deep sequencing showed higher levels of expression at night, despite a second peak shortly after lights on in the 2009 larval LD sample. In deep sequencing there were 6 reads in the day sample, and 10 at night for an LD ratio of 0.6, only just relevant via our 1.5 fold threshold (discussed above). Only time point 2 h had a significant difference (P<0.001); all other time points had no difference between LD and DD samples. Together these data show that this gene is transcribed in a circadian pattern under the control of a biological clock and probably plays a similar role as the *cycle/bmal* genes that play critical roles in clocks in other animals (see [14] for a review).

#### Eya

The *eyes absent* transcription factor *eya* has recently been shown to play a role in circadian processes and it, along with another transcription factor, *six*, have been implicated as key factors in regulating responses to changing day length [23], [24]. The closest match identified to *eya* in *Acropora* is NCBI EZ014333 (Figure S1). This gene is expressed at very low levels but displays strongest expression at night according to deep sequencing results, with a LD ratio of 0.17 (Table 1). Transcription is under the control of the endogenous light entrained clock, as the DD samples continued to show rhythmic cycling, with peak expression at night, although a second peak is present in the LD sample shortly after lights on (Figure 2e). Despite three time points having different expression levels (2 h: P<0.001, 14 h: P<0.05, 22 h: P<0.001), the one-way ANOVA confirmed no difference between LD and DD samples.

#### Timeless

*Timeless* (NCBI EZ013923) was expressed at highest levels in the dark (LD ratio 0.39) in two larval samples but does not show any diel rhythym under DD (Figure 2f). The interaction of light treatment and time was significantly different (P = 0.0057), as was the overall difference between light treatment (P = 0.0324) while time points 14 h (P<0.001), and 22 h (P<0.001), were significantly different between light treatments.

### Cyclic expression in adult tissues containing zooxanthellae

**Figure 3:**
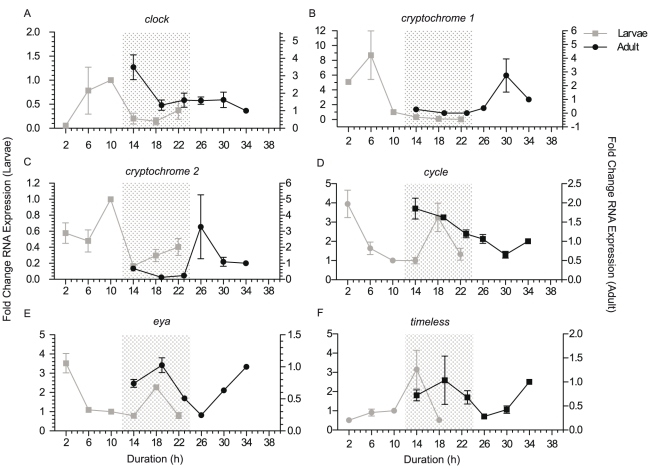
Comparison of larvae and adult diurnal gene expression. QPCR analysis of candidate circadian gene expression in larvae. Larvae and adult tissues were compared using QPCR, to determine similar patterns of gene expression over a 12∶12 LD treatment (a. *clock*, b. *cryptochrome 1*, c. *cryptochrome 2*, d. *cycle*, e. *eyes absent*, f. *timeless*). Relative fold changes in RNA expression levels (mean ± SEM of triplicate QPCR reactions) are presented for both 2009 larvae (primary y-axis) and adult tissue (secondary y-axis). Shaded areas represent the 12 hours of darkness for larvae (12∶12 LD) and 11 hours of darkness for adult tissue (13∶11 LD).

The data presented above were all obtained using larval mRNA, and in this species of coral, larvae lack zooxanthellae [26]. In adult tissues coral transcription may be influenced by the photosynthetic activity of zooxanthellae. To examine if the adult holobiont has similar cycles of transcription, adult tissue was collected and analyzed for expression of circadian genes by QPCR. Adult coral colonies were kept on a 13∶11 LD cycle, the same length as they had been exposed to on the reef prior to collection. Once again *RNA polymerase II* was used as a control, and all results independently confirmed using *adenosyl homocysteinase*. The results of this analysis are shown in Figure 3.

The six genes analyzed in adult tissues, *clock cry1*, *cry2*, *cycle*, *eya* and *timeless*, all displayed similar but altered patterns over their 13∶11 LD cycle as compared to their expression in 12∶12 LD cycle azooxanthallate larvae. Adult *cry1* (Figure 3b) displayed strongest expression 6 hours after lights on, mirroring both the pattern and timing of larval expression, although the fold change in the adult sample was a smaller scale. The four remaining genes displayed similar diurnal transcription curves in both larvae and adult, yet peaks and troughs were shifted by up to five hours. A three hour shift in peak *clock* expression was observed between larvae and adult (Figure 3a); larvae expression peaked at two hours prior to lights off, while in adult tissue it occurred one hour after lights off. A four hour shift in peak and trough expression patterns of *cry2* was observed (Figure 3c), with levels beginning to increase two hours after lights off in larvae, and six hours after lights off in adult tissue. Peak expression of *cycle* was observed during the night for both adult and larval samples (confirmed with deep RNA sequencing), and a peak shortly after lights on in larvae. Adult samples demonstrated highest expression levels one hour after lights off, and larval samples six hours, indicating a five hour shift. *Cycle* was the only gene that demonstrated earlier peaks and troughs of expression levels in adult samples compared to larvae (Figure 3d). *Eya* gene expression in larvae peaked six hours after lights off and the adult tissue also peaked at 6 hours lights off (Figure 3e). *Timeless* expression demonstrated a peak in larvae 2 hours after lights off while adult samples occurred six hours after lights off, demonstrating a four hour shift (Figure 3f). Most adult samples showed a larger fold change in expression levels compared to larvae. This may be due to the stronger light intensity to which adults were exposed (see methods).

## Discussion

Through the use of deep sequencing and QPCR, we have demonstrated diurnal patterns of circadian gene expression in the coral *Acropora millepora*. While all genes display distinct patterns throughout a 12∶12 LD period, only some can be classified as being regulated by a biological clock and showing similar cycles in both LD and DD experiments. *Clock*, *cryptochrome 2*, *cycle*, and *eyes absent* all continued rhythmic patterns of gene expression in the absence of light, while *cryptochrome 1* and *timeless* lost rhythmicity. *Clock* and both *cryptochromes* demonstrate higher expression during the day time, while *eya*, *cycle* and *timeless* have peak expression during the night.

Clock and light driven diurnal patterns of transcription both occur in 7 day old azooxanthallar larvae and in zooxanthallar adult holobiont tissue, though for most genes there is a shift in the time at which expression peaks in larvae versus adults. One possible reason for a shift in peaks of expression between larvae and adult is differences in sample light and dark exposure cycles. Larvae were on a 12∶12 LD cycle while adults were on a 13∶11 LD cycle; this shift in hours of daylight is correlated with an average shift in gene expression levels by up to five hours. As the number of daylight hours increases, the pattern of gene expression is expected to change as many circadian genes are entrained by light and participate in the transcription-translation feedback loop in response to the number of hours of sunlight exposure. With a longer exposure to light in the entrainment period, it is expected to see a later shift in peaks and troughs within samples. The intensity of white light also differed between the larvae and adult samples, and may have led to changes in amplitude and fold change. Adult samples had higher intensity of light (∼21,000 lux), while larvae were exposed to lower intensity (∼150 lux). Dim light entrainment and exposure can alter the amplitude of changes in circadian gene transcription, but periodicity is normally not affected [27], [28]. Other differences may also have been a result of differences between the holobiont adult tissue and azooxanthellae larval tissue.

Parts of this research confirm results by Reitzel et al. [11], Hoadley et al. [7] and Levy et al. [9], yet distinct differences are present from each of these studies. Levy et al. (2007) [9] found both *cry1* and *cry2* genes in *A. millepora* to become arrhythmic in constant darkness, acting only as light-regulated genes, yet results from our research suggests otherwise. While *cry1* confirms the previous report, *cry2* displays strong rhythmicity in constant darkness, indicating that, as in more complex animals [29], its transcriptional rhythm is driven by a light entrained biological clock in corals. Data by Hoadley et al. [7] also found elevated levels of *cry2* transcription levels in *F. fragum* under constant darkness in windows corresponding to daytime peaks, though the authors do not find these to be statistically significant they are clearly visible. Similarly, in the starlet sea anemone *Nematostella vectensis*, Reizel et al. [11] observed 2 to 3 times higher expression in the equivalent gene in the day window of animals kept under constant darkness ([11], Figure 3C). In each of these studies the transcription level of *cry2* under constant darkness was much less than observed in response to light, yet in our studies, at least for the first 24 hour cycle in full darkness, the peak in expression in subjective day is just as high under constant darkness (Figure 2).

Interestingly, there are considerable differences between the cycles of transcription reported here for *Acropora* and those described in *Nematostella* [11]. Unlike Reitzel et al. [11] we identified a clear light driven diurnal cycle of *timeless* expression, with peak expression during the early night, similar to patterns displayed in *Drosophila timeless* expression [30], while Reitzel et al. [11] showed only slight differences between different points in the light cycle in the anemone *timeout/timeless* gene.

The data for the *cycle* gene are also very different between organisms. In coral (this study), the *cycle* gene displays rhythmic expression in constant darkness, continuing to peak in DD at the same time as light treated samples (Figure 2), while in *F. fragum cycle* shows strongest and clock driven expression in the day [7]. In the anemone only minor diurnal differences were noted in both LD and DD samples [11]. Considering how well conserved networks tend to be in related organisms, these differences are surprising. The *Favia cycle*-like gene (NCBI AEH41598) is the most closely related gene to *Acropora cycle*, with the second closest being vertebrate *bmal*. It is possible that these genes are not orthologous, and these two studies are in fact investigating different but related genes, though this seems unlikely. It is more likely that the very small differences in *cycle* expression levels over time that were observed in *Nematostella* are not large enough to detect cyclic patterns accurately. It is also possible that the choice of control genes, and minor experimental differences, such as experimental light intensity or how well a specific primer pair works, might explain some of the differences. It is also possible that these animals do express some genes in different patterns.

*Clock* shows peak expression during late subjective day in *Nematostella* [11], similar to both *Acropora* [9] and *Favia* [7], and is confirmed with the results presented in this study. Despite these similarities, both *Nematostella* and *Favia clock* gene show little to no expression during constant darkness, while in this *Acropora* study, strong diurnal cycling in both LD and DD conditions are observed (Figure 2). This continued rhythmic expression in constant dark shows strong similarity to the *Drosophila clock* gene, which also continues cyclic expression in the absence of an entraining agent, although it shows peak expression in early morning rather than in the afternoon as we observe [31].

The deep sequencing data presented here serves as a powerful source of both controls for QPCR and a source for pathway analysis. It allows expression of low level abundance genes to be quantified, unlike microarray analysis. Deep sequencing also gives accurate quantitation and does not suffer from the variability introduced by differences in mRNA abundance, primer efficiency, magnesium optimization or other factors that can impact PCR based approaches. One of the drawbacks of this method is cost, which resulted in only two time points being sequenced. As different genes peak at different points in a 24 hour cycle the sequence analysis will be biased towards genes whose peaks or troughs happen to coincide with the selected samples.

Pathway analysis of the sequencing data was performed using GSEA [17], [18]. The presented clustering analysis is preliminary only. Sequencing of more samples and better annotation of the coral transcriptome will be necessary to perform an in depth analysis of differences between transcription under different light regimens. Gene ontology (GO) terms were selected for coral sequences by blasting *Acropora* contigs against the *Nematostella* genome. However, as the target genome has only been machine annotated and will contain many annotation errors, the GO terms attributed to coral transcripts will have a significant error rate that would be compounded by BLAST errors. Despite this caveat the clustering analysis did produce a interesting list of preliminary results showing diurnal differences in a number of pathways that have been previously described to display such cycles in other animals, for example mitosis [32], or pathways that make logical sense in an experiment such as ours- for example the activation of retinal metabolic processes and rhodopsin gene expression in the light phase [33]. A related study has recently been performed using microarrays by Levy et al. [8]. That study was performed in adult tissue containing zooxanthallae. Both our data from deep sequencing of azooxanthellate larvae and the zooxanthellate adult microarray data of Levy et al. [8] find the pathway showing the greatest difference between day and night to be the DNA photolyase gene set, despite the different sampling and clustering methodologies implemented. This is likely due to the presence of the cryptochrome genes within this group. Another pathway detected as activated in the daytime by both studies was mitochondrial respiration, and both studies also demonstrate that the cell cycle is regulated in a diel manner. Unfortunately the full cluster analysis of Levy et al. [8] is not published and more detailed comparisons of the different datasets will require future bioinformatic analysis. With better annotation and additional sequencing runs these types of approaches will allow accurate mapping of diel patterns of metabolism and be a powerful tool for understanding coral physiology.

In sum, our data shows that large changes in transcription occur over a 24 hour time period in corals when exposed to a 12∶12 or a 13∶11 light∶dark cycle. Some of the genes displaying diurnal transcription patterns correspond to genes known to regulate circadian processes in other animals. Some diurnal patterns are under the control of a light entrained endogenous clock and continue in constant darkness, while others respond directly to light. These results provide a basis for exploring how the genome contributes to the sensing and responses to time in corals and provide tools with which temporally regulated biological processes such as spawn timing can be dissected and understood.

## Supporting Information

Figure S1 ***Acropora eyes absent*** **aligned against** ***Drosophila eya*** **(NCBI NP_523492.1) E = 1e−21, 34/60 identities, 44/60 positives.** The reciprocal best match when searching the NCBI NR protein database are various vertebrate *eya1* homologs, such as mouse CAA07818.1 and human EAW86974.1 along with *Nematostella* XP_001629445.1. The closest match in nucleotide BLAST searches are *Nematostella* XM_001629395.1 (E 2e−27, 149/199 [75%] identity) and *Xenopus eya1* NM_001090419.1.
(EPS)

Table S1 QPCR primer sequences.
(DOC)

Table S2 Supporting Dataset: full analysis of GO clustering pathway analysis. Excel File.
(XLS)
