# Supplementary material for: Circadian Cycles of Gene Expression in the Coral, Acropora millepora
Source: PLoS One. 2011 Sep 19;6(9):e25072. doi: 10.1371/journal.pone.0025072 (PMC3176305; doi:10.1371/journal.pone.0025072)
Supplement: Table S1 — QPCR primer sequences. (DOC) [file pone.0025072.s002.doc]

Supplemental Table 1. QPCR primer sequences

| Name | Abbreviation | Forward Primer (5’ to 3’) | Reverse Primer (5’ to 3’) |
| --- | --- | --- | --- |
| *Candidate Circadian Genes* | | | |
| Cryptochrome1 | cry1 | ACTTAGCTCGCCATGCTGTT | GCCTGCATTCAGACTCCACT |
| Cryptochrome2 | cry2 | TGGCATTTAAGCTTGCCTCT | CATGCGCCAAACAGTTTTC |
| CLOCK | clk | ACTTGGCAGCCGTCATTTAC | CGCTTTGAGAGGCAAACATA |
| Cycle | cyc | GCCTTACTCCTGATGTTTCG | GGACCGGAGTTATGGAGTCT |
| Timeless | tim | AGTCCATTGTGCCATTTGAT | CAGCACTCTGTTGGTTCCTT |
| Eyes Absent 1 | eya1 | CtTGGCTGAcCTTGGCTTTA | AGTGGAAAAATTCCCCCAAG |
| *Reference Genes* | | | |
| RNA Polymerase II | RPII | CCAAACTCCAATCCACCTTG | AAGACCTAAATAGTCATCCATGAGG |
| Adenosyl homocysteinase | adoH | TACCCACACTACTTGGAAGGTATTA | CTGGGCATTTGAGTGATTTC |
